# Behavioral thermoregulation in *Locusta migratoria manilensis* (Orthoptera: Acrididae) in response to the entomopathogenic fungus, *Beauveria bassiana*

**DOI:** 10.1371/journal.pone.0206816

**Published:** 2018-11-28

**Authors:** Rouguiatou Sangbaramou, Ibrahima Camara, Xin-zheng Huang, Jie Shen, Shu-qian Tan, Wang-peng Shi

**Affiliations:** Department of Entomology and MOA Key Lab of Pest Monitoring and Green Management, College of Plant Protection, China Agricultural University, Beijing, China; Chinese Academy of Agricultural Sciences Institute of Plant Protection, CHINA

## Abstract

Insects such as locusts and grasshoppers can reduce the effectiveness of pathogens and parasites by adopting different defense strategies. We investigated the behavioral thermopreference of *Locusta migratoria manilensis* (Meyen) (Orthoptera: Acrididae) induced by the fungus *Beauveria bassiana*, and the impact this behavior had on the fungal mycosis under laboratory conditions. By basking in higher temperature locations, infected nymphs elevated their thoracic temperature to 30–32.6 °C, which is higher than the optimum temperature (25°C) for *B*. *bassiana* conidial germination and hyphal development. A minimum thermoregulation period of 3 h/day increased survival of infected locusts by 43.34%. The therapeutic effect decreased when thermoregulation was delayed after initial infection. The fungus grew and overcame the locusts as soon as the thermoregulation was interrupted, indicating that thermoregulation helped the insects to cope with infection but did not completely rid them of the fungus. A significant enhancement in the number of haemocytes was observed in infected thermoregulating locusts, reaching levels that were even higher than those observed in the controls. In contrast, haemocyte concentration was severely reduced in infected insects that did not thermoregulate. In infected non-thermoregulating locusts, the reduction in haemocyte number was accompanied by an increase in fungal blastospore concentration that was obvious in the haemolymph by day four. In contrast, no circulating blastospores were found in the haemolymph of infected thermoregulating locusts three days post-inoculation. We also examined the phagocytic activity of infected insects in vivo by using fluorescein isothiocyanate (FITC)-labelled silica beads. The proportion of beads that was engulfed by haemocytes in infected, thermoregulating insects was similar to that in the controls throughout the experiment, whereas the rate of phagocytosis in infected, non-thermoregulating insects progressively decreased after infection. These findings demonstrated that behavioural thermoregulation can adversely affect *B*. *bassiana* mycosis in infected *L*. *migratoria manilensis*, thereby limiting the development of lethal entomopathogenic fungi in locusts. This is apparently accomplished through an increase in the levels of haemocytes, leading to greater phagocytic activity under certain environmental conditions.

## Introduction

A dozen subspecies of the migratory locust, *Locusta migratoria* (Orthoptera: Acrididae) have been recorded from various parts of the world, all of which are major pests in agriculture, causing considerable economic loss [1–4]. Under favorable environment conditions, certain locust species exhibit gregarious and migratory behaviour, leading to the formation of spectacular swarms [5]. In some countries such as Argentina, Australia, China, Niger and South Africa, population of locusts and grasshoppers are treated as soon as outbreaks threaten [5]. In China, 1.5 to 3 million ha are infested each year by *L*. *migratoria manilensis* (Meyen) and, as a result, management of the locust has received considerable attention for many years [6]. Chemical pesticides have traditionally been the most effective control method against locusts; however, this practice has been shown to lead to serious environmental pollution, as well as entailing high costs for emergency control [5]. To mitigate the serious environmental problems that have resulted from the continuing overuse of chemical pesticides, biopesticides including the microsporidian *Paranosema* (*Nosema*) *locustae* and the entomopathogenic fungi *Metarhizium acridum* and *Beauveria bassiana* have been increasingly employed to control locust outbreaks [6–11]. Their use also helps avoid chemical residues in agricultural products as well as in environmentally sensitive areas, such as wetlands [12]. Entomopathogens are not only capable of killing large numbers of locusts under overcast conditions [5,6,13,14], but they can also alter their morphological phase transformation [9,14], forestalling the formation of dense bands and locust swarms [15].

The entomopathogenic fungus, *Beauveria bassiana* has frequently been reported as an effective biological control agent of locusts [16–18] and has been successfully tested in a number of formulations as a grasshopper mycopesticides in Canada and the USA, and, as a ULV application in Mali [5,18]. Nevertheless, field studies have shown that the fungus is relatively slow and variable in inducing mortality [19–22]. In addition, insects have managed to evolve numerous defensive strategies to overcome pathogen or parasite infections [23–25]. One of these defences is thermoregulation (i.e., behavioral fever), an increase of body temperature in infected insects through behavioral changes [26,27]. Fever is an effective means of reducing pathogen virulence and prolonging the survival time of infected insects because it is a component of immune defense. It can also enhance other aspects of immune defense [28,29]. Host thermal biology can cause fungi to be less effective. This phenomenon has been reported in several hosts in various insects orders, including Hymenoptera (pathogens: *Ascosphaera apis* and *Nosema ceranae*) [30,31], Diptera (pathogens: *Entomophthora muscae* and *E*. *schizophorae*) [32], and Orthoptera (pathogen: *M*. *anisopliae* var. *acridum*) [33–35].

In Orthoptera, many species of locusts, grasshoppers, and crickets have the capacity to develop a behavioural fever [34,36]. Infected desert locusts, *Schistocerca gregaria* (Forskål), can elevated their body temperature to as much as 38–40 °C, a range which is detrimental to the growth of *M*. *anisopliae* var. *acridum* [21], and above the upper limit for *B*. *bassiana* growth and infection [37]. Such thermoregulation (through basking) reduced mortality 35% in infected *S*. *gregaria* that were allowed to thermoregulate [33]. Thermoregulation has been shown to play a significant role in host–pathogen associations [26,38,39,40]. Although thermoregulation had been shown to play a crucial role in a number of infected insects [20], to date, research has not been focused on *L*. *migratoria manilensis* inoculated by *B*. *bassiana*. The optimal temperature for germination and growth of the fungus *B*. *bassiana* ranges from 25–30°C [37] which is typically below the body temperature of thermoregulating locusts.

In addition to its role in improving host survival, changes in preferred body temperature in relation to environmental temperatures had been found to correlate with an increase in levels of host immunity [38,40]. The innate immune system of insects comprises cellular and humoral responses [41], which act complementarily to control the spread of a disease. Humoral components are the release of molecules such as antimicrobial peptides, proteins and enzymatic cascades that regulate the formation and clotting of melanin [42,43,44,45], although these processes require an extended period of time to function. In contrast, cellular components respond immediately when invasion by foreign materials occurs. These cellular components of defense consist of responses such as phagocytosis, nodulation, and encapsulation, which are mainly performed by haemocytes [46,47]. Previous studies have shown that elevation of insect body temperatures can induce changes in some host behaviors [26,48], as well as initiate responses in the insects’ immune performance, such as an increase in the enzymatic activity of phenoloxidase and lysozyme-like enzymes, while holding haemocytes numbers steady [38,40,49]. Although there is increasing evidence of the importance of host thermal biology in host-pathogen interactions (such as the influence of thermoregulation on insects disease, as well as the immune response) [26,38,40], no research has yet been conducted on the oriental migratory locust, *L*. *migratoria manilensis*, infected by the fungus *B*. *bassiana*. In this study we determined the effects of environmental temperature and host thermal biology on *B*. *bassiana* efficacy. We also assessed the influence of thermoregulation on host internal defenses by recording changes in numbers of haemocytes and blastospores in the host haemolymph. The level of phagocytic activity in infected and uninfected *L*. *migratoria manilensis* under thermoregulation and non-thermoregulation condition was monitored using fluorescent silica beads [50]. These results will help improve strategies when incorporating fungal biopesticides in integrated locust management by overcoming the effects of induced fever in locusts.

## Materials and methods

### Sources and rearing of locusts and pathogens

The Oriental migratory locusts, *Locusta migratoria manilensis*, used for this study were obtained from a laboratory colony maintained by the Key Bio-control Laboratory for Locusts at the China Agricultural University, Beijing. Nymphs were reared in cages (44 × 18 × 15 cm) in an artificial climate incubator at 30 ± 1°C and 60 ± 10% RH and a 16:8 h L:D photoperiod. Insects were fed with wheat leaves, and cages were cleaned daily.

The *B*. *bassiana* strain was obtained from the same laboratory. It was originally isolated from dead locust collected from a grassland of Qinghai Province, China. The strain was kept in the lab at -70 °C for several years. *B*. *bassiana* was activated on potato dextrose agar (PDA) in an incubator at 27 °C for 14 days to allow complete sporulation. Spores were collected by flooding the Petri dishes with 2 × 5 mL of a sterile solution of 0.05% Tween 80 and then gently raking the dish with a sterilized curved bar to release spores. The spores were harvested from the resulting suspension by centrifugation at 4 °C for 10 min (10,000 *g*). A haemocytometer was used to measure the spore concentration, and sterile distilled water was used to dilute the mix to the desired concentration. The final solution was then held at 4 °C until used. Fifth instar locust nymphs were used in all experiments. Insects were injected with 1 μL of the fungal solution into the thorax using a 5 μL micro syringe, except for the experiment on delaying and interruption thermoregulation, in which infected insects were inoculated (contact) with 2 μL of fungus solution. Control insects were injected with an equal amount of sterile distilled water.

### Behavioural thermoregulation

To determine the impact of *B*. *bassiana* infection on the internal body temperatures of *L*. *migratoria manilensis*, 14 locusts (seven males and seven females) were maintained in cages similar to those described above at a controlled room temperature of 30 ± 1 °C and 60 ± 10% RH. Each cage was equipped with a 40 W incandescent lamp which produced a vertical heat gradient, allowing insects to voluntarily move up and down to regulate their body temperature. The bulbs were switched on for 10 h per day (from 8:00 AM to 6:00 PM).

The internal body temperatures of *L*. *migratoria manilensis* were recorded using a handle thermocouple (OMEGA, HHFB1). The tip of the thermocouple was sterilised with 75% alcohol before insertion into the insect’s thorax, and the temperature of each insect was recorded for 5–10 seconds. Each locust in the treatment group was inoculated with 1 μL of the fungus *B*. *bassiana* at 10^5^ spores/mL. Control insects were injected with sterile distilled water. The body temperatures of locusts were monitored every day at 8:00 am, 10:30 am, 1:00 pm, 3:30 pm and 6:00 pm (five observations/day) for 10 days following fungal inoculation.

### Activity record

To determine the effect of *B*. *bassiana* infection on the feeding pattern of *L*. *migratoria manilensis*, the distribution and activity of insects were monitored. Locusts were inoculated with 1 μL of 10^4^ spores/mL of the fungus solution, and control insects were injected with the same volume of sterile distilled water. Insects were then distributed into cages equipped with a 40 W incandescent lamp. The incandescent bulbs were turned on for 6 h each day (from 8:00 AM to 2:00 PM). The experiment was performed under controlled conditions at 30 ± 1 °C and 60 ± 10% humidity and 16:8 h L:D photoperiod. To monitor locust activity, each cage was arbitrarily divided into three sections. The upper section of the cage was the basking zone (B) with temperature above 30 °C, the middle section of the cage was considered as the resting zone (R), and the bottom part was the feeding zone (F) where leaves were continuously provided. Insects were able to bask at will and had continuous access to food. Observations were performed for 6 days after inoculation. Ten locusts per cage were used for each treatment, and the experiment was repeated three times (n = 30 for three biological replicates).

### Varied duration of thermoregulation

Insects inoculated with 1 μL of 10^4^ spores/mL of *B*. *bassiana* were kept in cages as described above and assigned to three different thermoregulation regimes: 0 h, 3 h and 6 h per day. The 0 h regime consisted of infected locusts that were not allowed to thermoregulate. Control insects were injected with sterile distilled water. The experiment was performed under conditions identical to those described above (Behavioral thermoregulation). Ten locusts were placed in a single cage for each treatment, and each experiment was conducted three times (n = 30 for three biological replicates). Tests lasted for 20 days after inoculation, and mortality was checked daily. Dead insects were disinfected with 75% alcohol and washed with sterile distilled water. Samples were then placed into Petri dishes containing moistened filter paper and put into an incubator at 27 °C for five days to detect *B*. *bassiana* sporulation from the cadavers.

### Delaying thermoregulation

To examine the impacts of delaying thermoregulation on locust survival, we exposed insects to three different post-inoculation thermoregulation regimes. Insects were allowed to initiate thermoregulation 0, 24, or 48 h after inoculation. The thermoregulation period lasted for seven h per day. Infected insects under non-thermoregulation conditions were considered the controls. Insects were inoculated per contact in the thorax with 2 μL of 10^6^ spores/mL. Twenty-five locusts were kept in each cage and each treatment was replication three times. The experimental conditions were identical to those in the “varied duration of thermoregulation” experiment above.

### Interruption of thermoregulation

In this experiment, we ended the thermoregulation to determine its effect on the *B*. *bassiana* mycosis. Thermoregulation (7 h/days) was interrupted 11 d post-inoculation for infected insects that had initiated thermoregulation 0, 24, and 48 h after inoculation. Mortality was recorded for 10 days following thermoregulation interruption. Deceased insects were processed as described above.

### Sample collection to measure locust immune response

In order to assess the immune response of *L*. *migratoria manilensis*, locusts were inoculated with 1 μL of 3×10^5^ spores/mL of *B*. *bassiana* and kept in cages under the same controlled conditions as described above. Insects were exposed to two regimes: with thermoregulation (TR) and without thermoregulation (NTR). A thermal gradient was provided by a 40 W incandescent light hung above each cage and turned on for seven h per day. To determine fungal blastopore and locust haemocyte concentrations and to check for the strength of the locust’s phagocytic activity, a 5 μL syringe was inserted into the trochanter of the insects, and a small aliquot of haemolymph was quickly pipetted into an ice-cold solution consisting of one part of CEB (citrate-EDTA buffer, 69 mM KCL, 27 mM NaCL, 2 mM NaHCO_3_, 100 mM d(+)–glucose, 30 mM tripotassium citrate, 26 mM citric acid, and 10 mM Na_2_-EDTA, pH4.6) and one part of Grace’s Insect Medium (GM) (Gibco) [51]. Prior to the experiment, Eppendorf tubes and pipette tips were sterilized and pre-cooled, and the syringe insertion point and syringes themselves were disinfected with 75% alcohol.

### Haemocytes and fungal blastospores counts in haemolymph samples

Haemocytes concentration was assessed daily for five days in samples collected as described above. The four treatments consisted of uninfected non-thermoregulating (CNTR), uninfected thermoregulating (CTR), infected non-thermoregulating (INTR), and infected thermoregulating (ITR) locusts. Following infection, a minimum of 10 insects were individually processed daily for each treatment. To determine the total haemocyte count (THC) and blastopore count, 20 μL of haemolymph were collected per sample and gently mixed with 200 μL of buffer solution (described above) and observed using a haemocytometer under a phase contrast microscope (400×). Fifty μL of the remaining haemolymph in each sample was then plated out on Dichloran Rose Bengal Chloramphenicol DRBC (31.6 g/L) and held at 27 °C for five days to detect any fungal bodies that may have been present in the haemolymph sample that could not be observed under the microscope.

### Phagocytic activity

To evaluate the level of phagocytic activity in infected locusts, fluorescein isothio-cyanate (FITC)-labelled silica beads were used to visualize the response and count the number of engulfed or non-engulfed beads [50]. Silica beads were first sterilized in 75% alcohol and then suspended in a sterile solution of carbonate-bicarbonate (9.5 mL of 0.2 M Na_2_CO_3_, 41.5 mL of 0.2 M NaHCO_3_, and 150 mL H_2_O; at pH 9.4). One milligram of FITC powder was added per mL of the bead suspension, and the mixture agitated for 30 min at room temperature in complete darkness. After agitating, fluorescent beads were recovered following serial washing (centrifugation at 500 g, for 10 min) using a phosphate buffer solution (8 g NaCl, 0.2 g KCl, 1.44 g Na_2_HPO_4_, 0.24 g KH_2_PO_4_/L; pH 7.2) [50]. Beads were then resuspended in Grace’s Insect Medium and the concentration adjusted to 10^6^ mL using a haemocytometer. Two μL of the bead suspension were then injected into locusts that were either inoculated and not inoculated with fungus. At a preselected time post-injection, haemolymph samples were collected (as described previously) to measure the proportions of engulfed and non-engulfed beads. Twenty μL of haemolymph was carefully mixed with an equal volume of CEB/GM (see above) and drops of the mixture were then placed on a haemocytometer to check for engulfed versus non-engulfed beads. Using an OPTES CCD TP310 microscope equipped with an ultra-fine colour engine, a total of 100 beads were recorded from a given field of view (400×). If necessary, additional fields of view were randomly selected on the slide until 100 beads had been recorded. Phagocytic activity of insects was assessed only by counting haemocytes that had engulfed beads [52]. Trypan blue (2 mg/mL) was used as the quencher. Beads were injected daily, and haemolymph samples were collected daily three h after injection to measure the phagocytic activity of infected locusts over the course of the disease. The experiment was conducted for five consecutive days with 8–10 samples checked per treatment.

### Statistical analysis

All experiments were arranged as a randomized block (cage) design, and the repartition of insects in cages was analysed using a one-way ANOVA for the behavioral thermoregulation, varied duration, delaying and interruption of thermoregulation, haemocyte and fungal blastospore counts, and phagocytic activity. Independent-samples T-tests were used to test for the activity record. Treatments were compared using the Fisher least significant difference (LSD) test (α = 0.05). The software IBM SPSS 24 was used for the analyses.

## Results

### Infected locusts elevate their body temperature through behavioral selection of high temperatures

**Fig 1:**
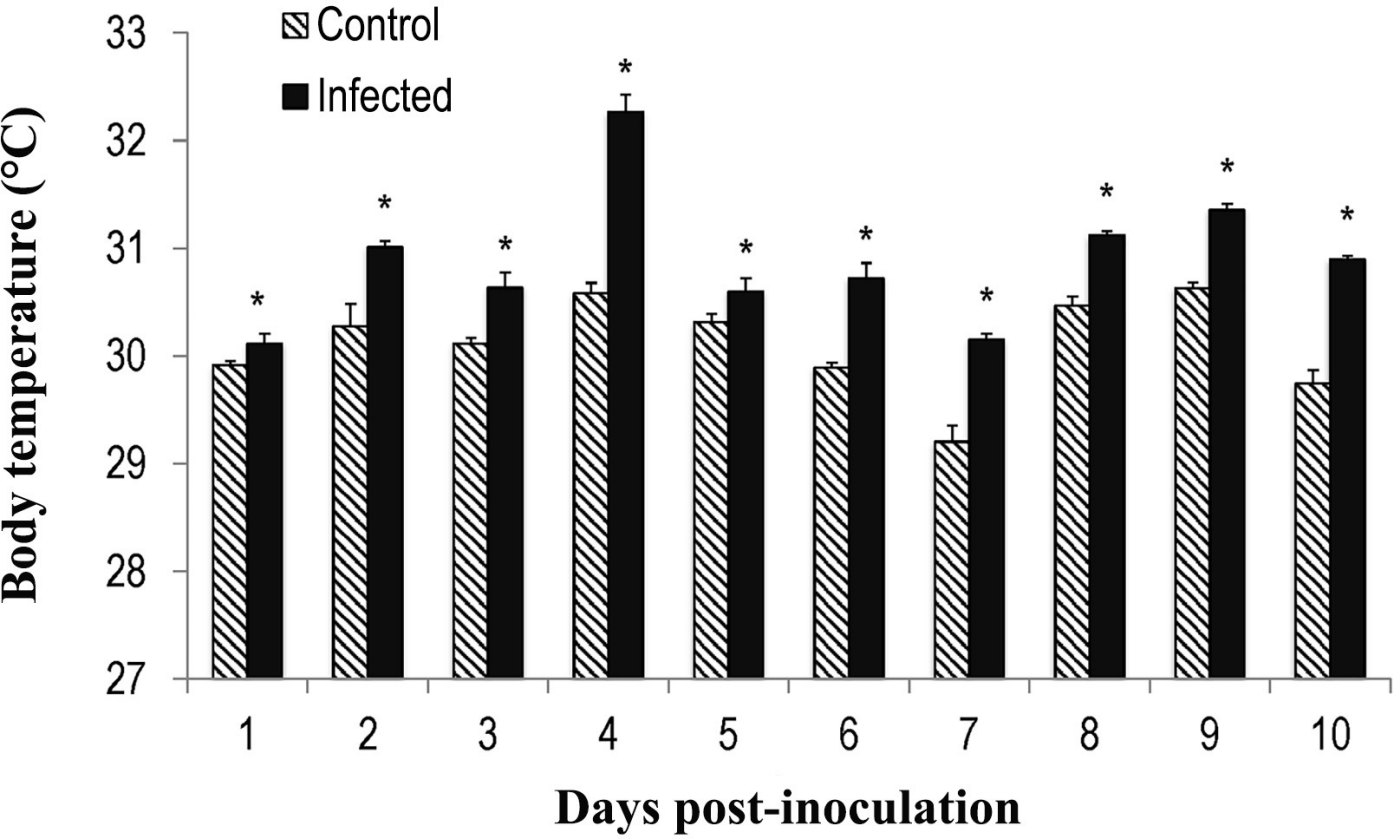
Body temperatures of *Locusta migratoria manilensis* after infection with *Beauveria bassiana*. Comparison of the mean daily body temperatures of *L*. *migratoria manilensis* inoculated with 10^5^ spores of *B*. *bassiana* /5^th^ instar nymph, and the controls (inoculated with sterile distilled water). Asterisks denote a significant difference in body temperature between infected and non-infected insects (*P* < 0.05).

While control locusts maintained a constant body temperature, *B*. *bassiana*-infected locusts had elevated body temperatures during the entire 10 days, with an increase of 1–3 °C relative to healthy individuals held under an identical rearing temperature (30±1°C). The highest body temperature recorded was 32.6 °C at day 4 d post-inoculation (Fig 1). The differences in body temperatures were significant between inoculated and control insects on each of the post-infection days measured: day 1 (*F* = 16.526, *P*< 0.05), day 2 (*F* = 16.450, *P*< 0.05), day 3 (*F* = 42.401, *P* < 0.05), day 4 (*F* = 37.382, *P* < 0.05), day 5 (*F* = 25.006, *P* < 0.05), day 6 (*F* = 31.056, *P* < 0.05), day 7 (*F* = 19.880, *P* < 0.05), day 8 (*F* = 46.374, *P* < 0.05), day 9 (*F* = 50.170, *P* < 0.05) and day 10 (*F* = 41.624, *P* < 0.05).

### Alteration of basking and feeding behaviors in infected locusts due to thermoregulation

**Fig 2:**
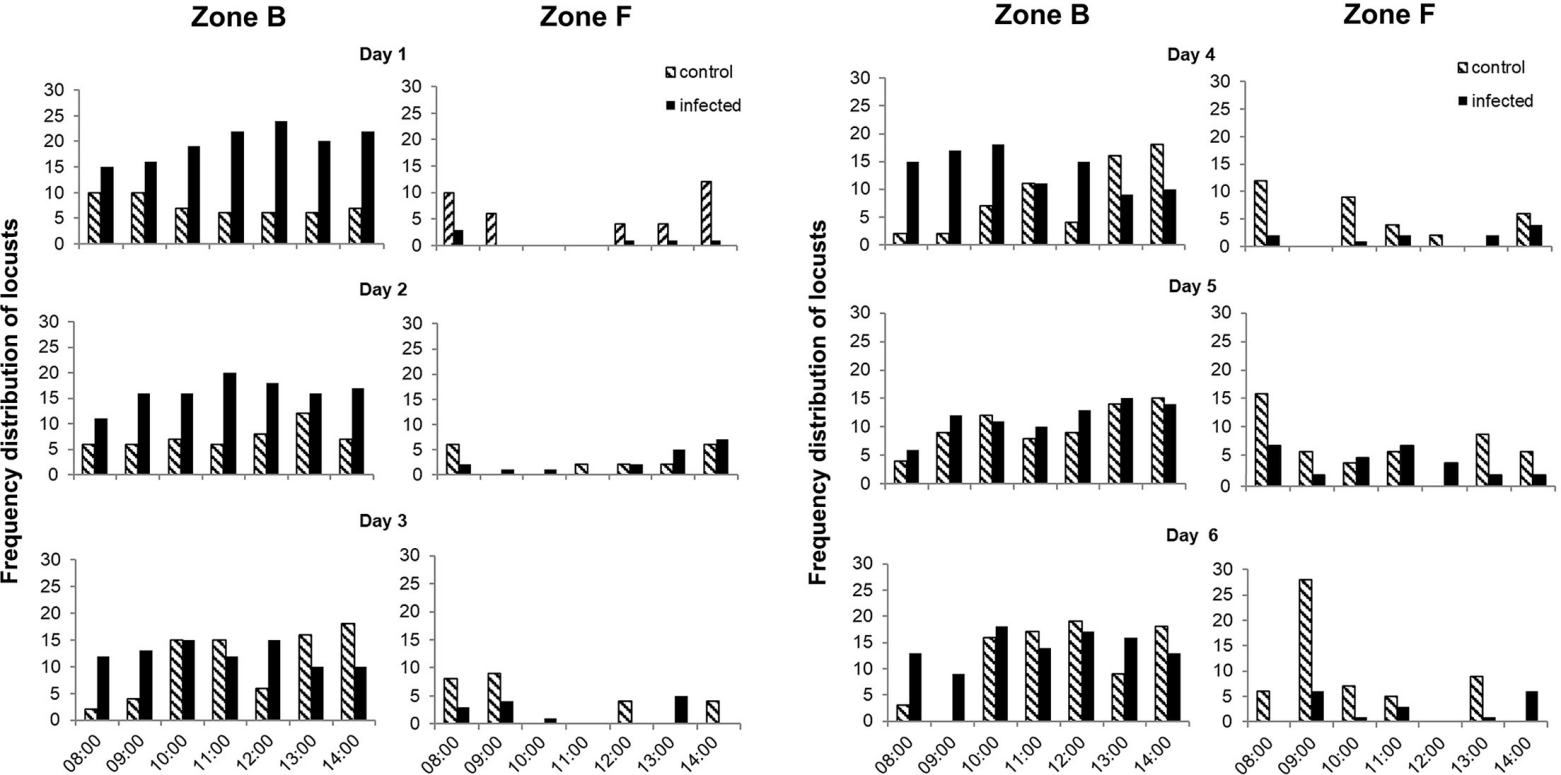
Feeding and basking behavior of infected *Locusta migratoria manilensis*. Frequency distribution of *L*. *migratoria manilensis* in zone B (basking) and zone F (feeding) in cages providing a heat gradient. Infected locusts were inoculated with *Beauveria bassiana* (10^4^ spores/ 5^th^ instar nymph). n = 30 in three biological replicates.

**Fig 3:**
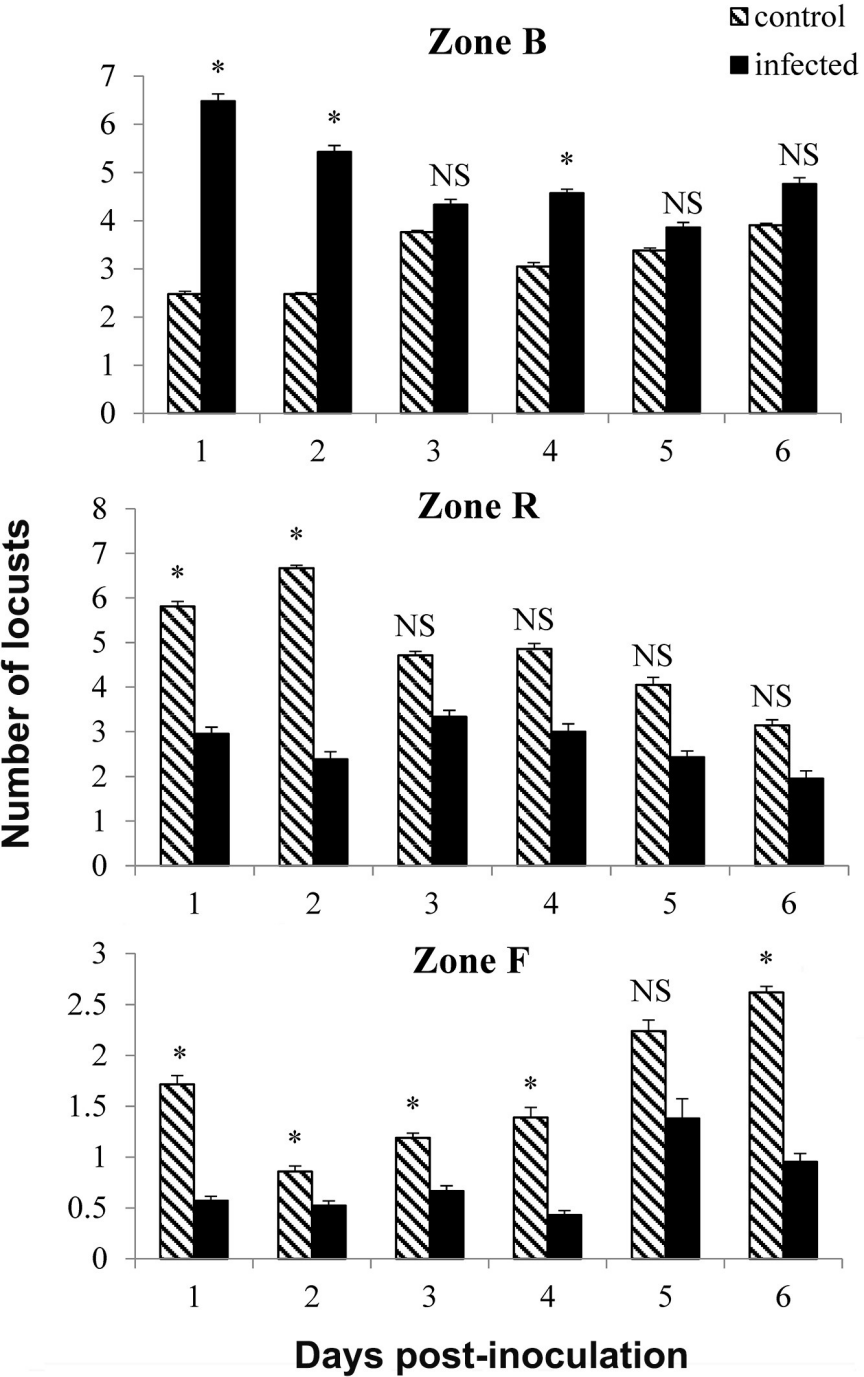
Distribution of infected and uninfected locusts in feeding, basking and resting zones. Distribution of control and inoculated *Beauveria bassiana*-infected *Locusta migratoria manilensis* (10^4^ spores/5^th^ instar nymph) in zone F (feeding), zone B (basking) and zone R (resting). An asterisk denotes a significant difference between infected and non-infected insects (*P* < 0.05), and NS indicates no significant difference. n = 10 locusts per treatment.

The distribution frequency of infected locusts was greatly increased relative to the controls in zone B (basking zone) after the light was turned on (Fig 2). Significant differences were found in half of the test days: day 1 (*F* = 6.250, *P* = 0.002), day 2 (*F* = 7.652, *P* = 0.008), and day 4 (*F* = 0.727, *P* = 0.000), but no differences were noted on day 3 (*F* = 10.562, *P* = 0.222), day 5 (*F* = 3.122, *P* = 0.298), or day 6 (*F* = 1.926, *P* = 0.236). Control insects showed a tendency to feed more than the infected locusts, although the differences were not significant on day 2 (*F* = 4.000, *P* = 1.000) or day 5 (*F* = 5.199, *P* = 0.377). Feeding differences were significant on day 1 (*F* = 0.000, *P* = 0.004), day 3 (*F* = 2.571, *P* = 0.013), day 4 (*F* = 5.953, *P* = 0.036), and day 6 (*F* = 8.471, *P* = 0.010) (Fig 3).

### Varied time of thermoregulation regime influenced efficacy of *B*. *bassiana*

**Fig 4:**
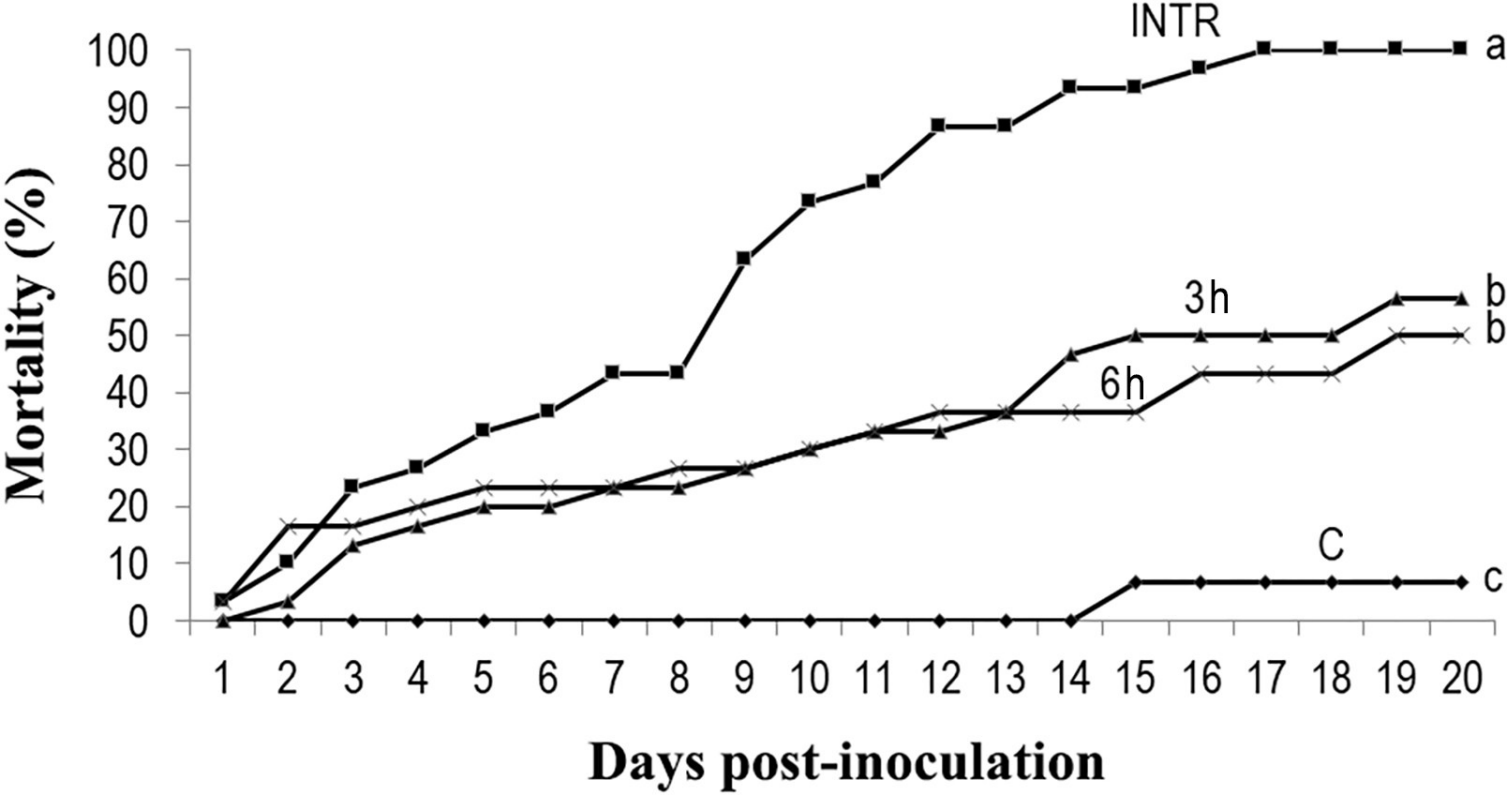
Impact of thermoregulation duration on mortality of infected locusts. The cumulative mortality of *Locusta migratoria manilensis* inoculated with *Beauveria bassiana* (10^4^ spores/5^th^ instar nymph) following exposure to three regimes of thermoregulation (0 h, 3 h and 6 h per day are indicated by filled squares, triangles and Xs, respectively). The 0 h regime corresponded to inoculated non-thermoregulating locusts (INTR). C-non-infected, thermoregulating locusts are indicated by filled rhombuses. (n = 30 for three biological replicates). ANOVA on mortality on day 20 is followed by LSD (different letters indicate significant differences).

All infected non-thermoregulating insects had died within 17 days post- infection, with 50% of mortality occurring within 9 days post-infection. Mortality was reduced in thermoregulating insects, but remained significantly higher than it was in the control individuals (Fig 4). Survival distribution curves were significantly different between treated and control locusts (*X*^*2*^ = 10.66, *df1* = 3, *df2* = 8, *P* = 0.004). Fungal infection was evident in 67% of the inoculated, non-thermoregulating dead locusts, while 41% of inoculated locusts experiencing under 3 h of thermoregulation, and 26% under 6 h of thermoregulation showed evidence of fungal infection. No *B*. *bassiana* sporulation was found in the untreated insects during the experiments.

### Delaying thermoregulation increased efficacy of *B*. *bassiana*

**Fig 5:**
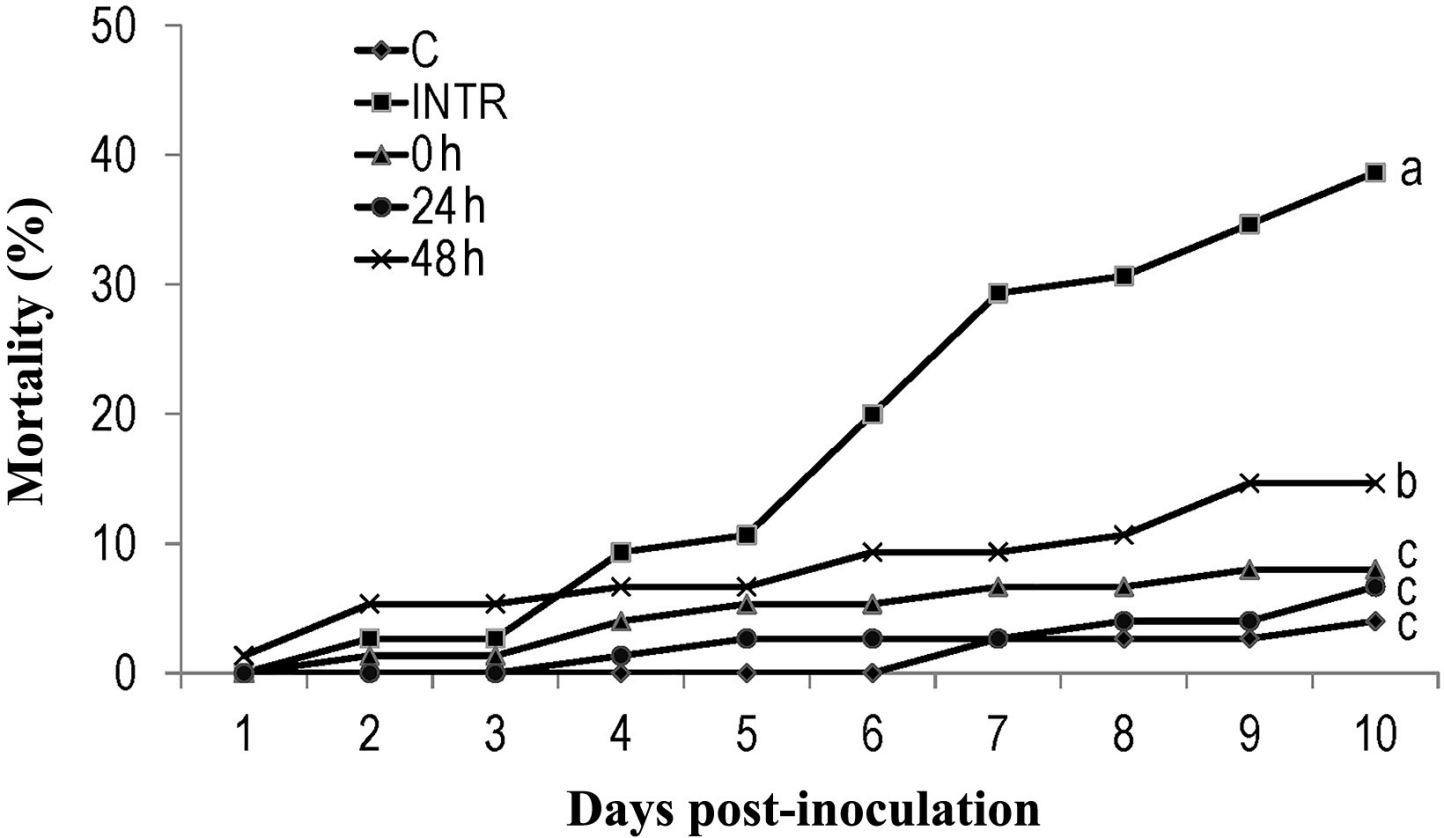
Effect of thermoregulation onset on mortality of infected locusts. Mortality of *Locusta migratoria manilensis* inoculated with *Beauveria bassiana* (10^6^ spores/5^th^ instar nymph) and exposed to various thermoregulation durations (0 h, 24 h and 48 h post-inoculation are indicated by filled squares, triangles, circles and Xs, respectively). The 0 h regime corresponds to infected locusts allowed to thermoregulate immediately after infection. INTR indicates inoculated and non-thermoregulating locusts. C indicates non-thermoregulating and non-infected control locusts. n = 25 insects per treatment. ANOVA on mortality at day 10 is followed by LSD (different letters indicate significant differences).

The mortality of locusts with early access to thermoregulation (0 h and 24 h after inoculation) was significantly lower than in locusts forced to delay thermoregulation until 48 h after inoculation (Fig 5). The survival distribution curves were different among these thermoregulating treatments (*X*^*2*^ = 5.27, *df1* = 3, *df2* = 8, *P* = 0.02).

### Interruption of thermoregulation increased locust mortality

**Fig 6:**
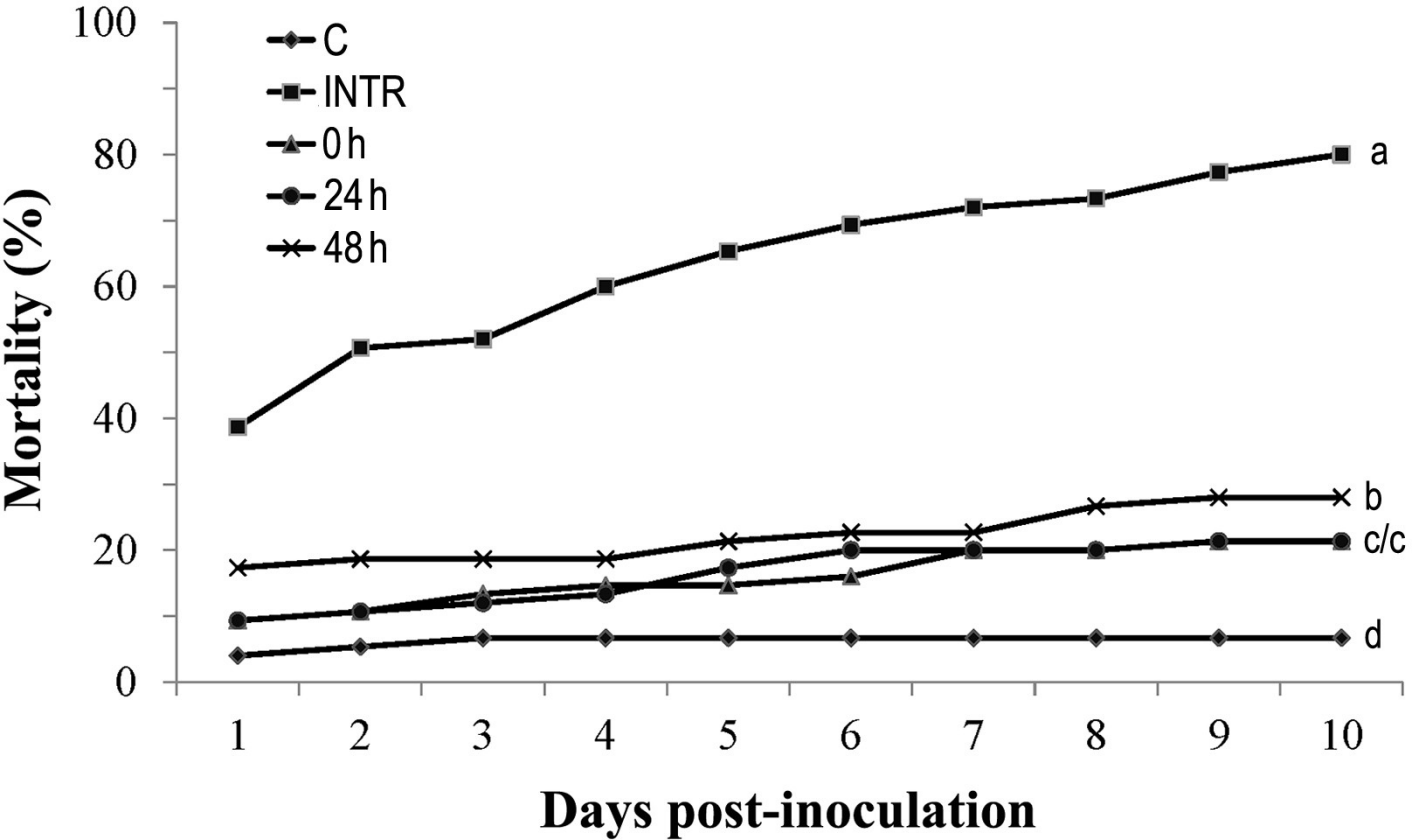
Impact of early termination of thermoregulation on the survival of infected locusts. The mortality of *Locusta migratoria manilensis* inoculated with *Beauveria bassiana* (10^6^ spores/5^th^ instar nymph) after early termination of thermoregulation. Insects were initially allowed to thermoregulate (0, 24, or 48 h post inoculation, with INTR as a control). The opportunity to thermoregulate was ended after 10 days (so that day 11 became day 1 of post-interuption, as labeled in the figure). C represents non-thermoregulating and non-infected control locusts. INTR represents inoculated and non-thermoregulating locusts, with n = 25 insects per treatment. ANOVA was conducted on mortality at day 10 followed by LSD (different letters indicate significant differences).

As thermoregulation ceased, the survival rate of infected locusts decreased thereafter, while remaining constant in the control locusts (*F* = 6.884, *df* = 3, *P* = 0.013; Fig 6). The increase in mortality of locusts allowed to initiate thermoregulation 48 h after inoculation began six days post-inoculation, and reached 28% by the end of the experiment. This increase in mortality occurred at a similar rate for the 0 h and 24 h treatments. The mortality rate of infected non-thermoregulating insects was 80% by the tenth (and last day) of the experiment, which differed significantly from all thermoregulating treatments. Survival distribution curves were significantly different among the 0, 24 and 48 h treatments (*X*^*2*^ = 3.96, *df1* = 3, *df2* = 8, *P* = 0.05) (Fig 6).

### Haemocyte and blastospore count

**Fig 7:**
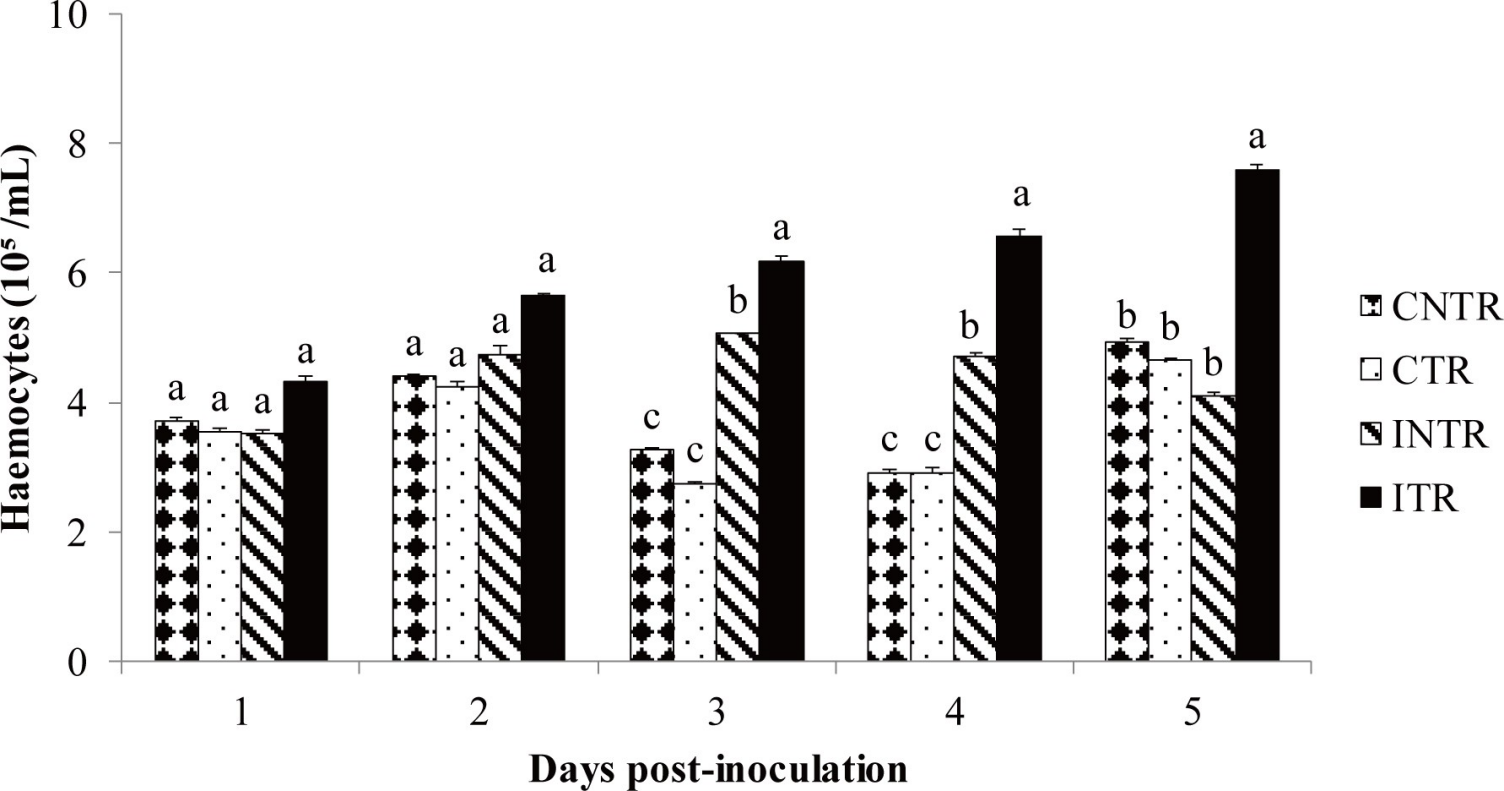
Total haemocyte count. Mean haemocyte concentrations in haemolymph samples of *Locusta migratoria manilensis* inoculated with *Beauveria bassiana* (3×10^5^ spores/5^th^ instar nymph) at different days post-infection. CNTR, control non-thermoregulating insects; CTR, control thermoregulating insects; INTR, infected non-thermoregulating insects; ITR, infected thermoregulating insects. Data are given as concentration (means ± SE). 10–15 insects were infected per treatment and per interval. Different letters indicate significant differences.

**Fig 8:**
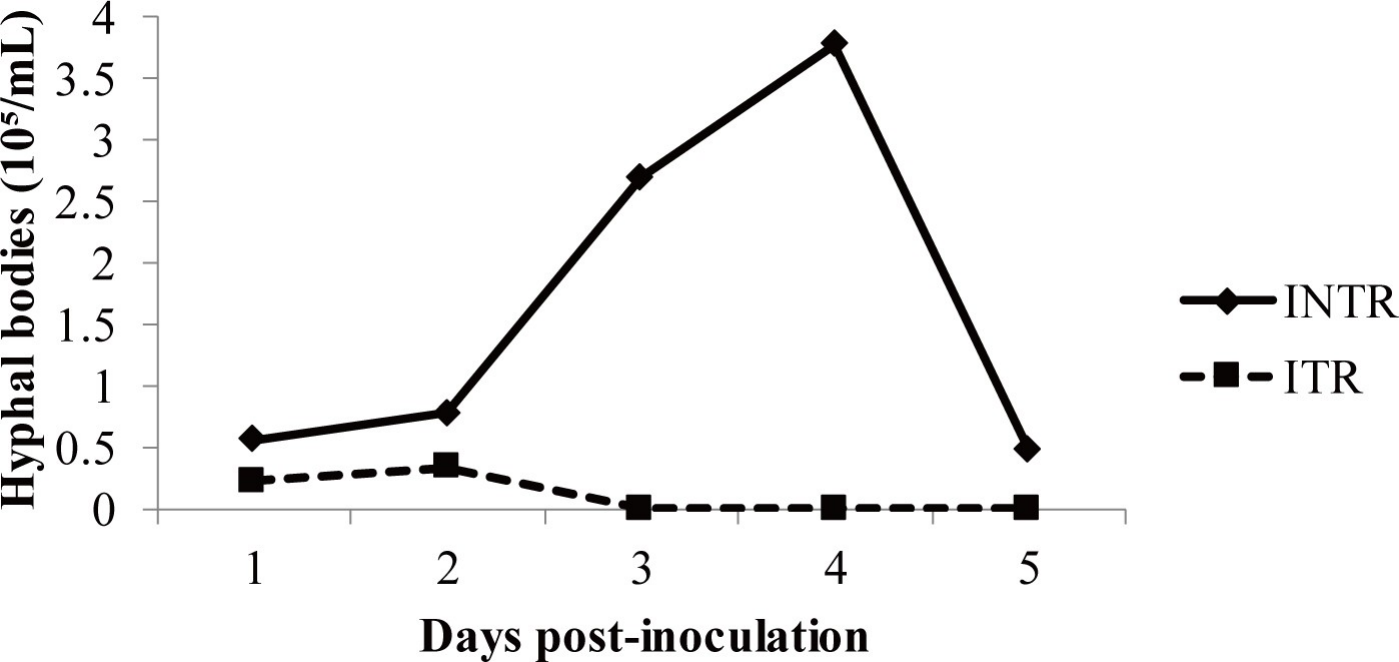
Total blastospore count. Mean blastospore concentrations in haemolymph samples of *Locusta migratoria manilensis* inoculated with *Beauveria bassiana* (3×10^5^ spores/5^th^ instar nymph), at different numbers of days post-infection. INTR, infected non-thermoregulating insects; ITR, infected thermoregulating insects. Data are given as concentration (means ± SE). 10–15 insects were infected per treatment and per interval.

Prior to fungal inoculation, the haemocyte concentration of locusts averaged approximately 9×10^4^ cell/μL with several types of haemocytes present. Granulocytes and plasmatocytes were the most common cells present in the haemolymph. The number of haemocytes in non-infected control locusts was not influenced by thermoregulation, but was in fungal infected locusts. Haemocyte concentrations were not significantly different between the control and the infected locusts for the first two days after inoculation (day 1: *F =* 0.679, *P =* 0.581; day 2: *F =* 0.843, *P =* 0.496), after which, in days 3 to 5, there was a substantial increase in haemocyte concentration in infected thermoregulating locusts that was statistically different from infected non-thermoregulating locusts, as well as the uninfected control locusts (day 3: *F* = 30.131, *P =* 0.000); day 4: *F =* 11.072, *P =* 0.001 and day 5: *F =* 9.787, *P =* 0.002). Indeed infected, non-thermoregulating locusts had haemocyte levels that were higher than control locusts only on days 3 and 4; thereafter, haemocyte levels decreased until they were not statistically different from those of the control insects by day 5 (Fig 7). Haemocyte concentrations in infected, non-thermoregulating locusts started to decline on day 4, coinciding with an increase in the density of blastospores in the haemolymph (Fig 8). Circulating blastospores were observed in thermoregulating insects only on days 1 and 2 post-infection, and were absent in subsequent days. In addition, the count of colony forming units (CFUs) on selective media demonstrated that there were fewer circulating blastospores in infected locusts that were allowed to thermoregulate than in those locusts that were not. However, a decrease in the numbers of circulating blastospores was observed in non-thermoregulating locusts on day 5, which were apparently resistant to the infection.

### Phagocytic activity

**Fig 9:**
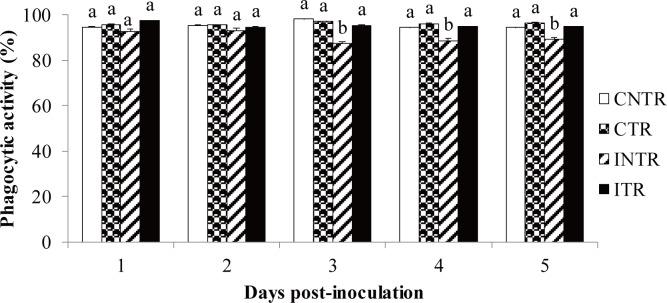
In vivo phagocytosis. In vivo phagocytic activity of *Locusta migratoria manilensis* inoculated with *Beauveria bassiana* (3×10^5^ spores/5^th^ instar nymph) and injected with FITC-labelled silica beads at various days post-infection. CNTR means control non-thermoregulating insects; CTR, control thermoregulating insects; INTR, infected non-thermoregulating insects; and ITR, infected thermoregulating insects. Data are given as percentages (means ± SE). 8–10 insects were used per treatment and per interval. Different letters indicate significant differences.

Fluorescein isothiocyanate-labelled silica beads were engulfed by both round and spindle-shaped haemocytes. The number of engulfed beads did not differ between uninfected and infected locusts on days 1 or 2 post-infection (day 1: *F =* 1.731, *P =* 0.238; day 2: *F =* 0.461, *P =* 0.717). However, that proportion decreased significantly in infected, non-thermoregulating locusts as infection time increased (Fig 9). By day 3, the average number of phagocyte cells was 87.25 ± 4.88% (*X*^*2*^ *= 5*.*520*, *df1 = 3*, *df2 = 8*, *P = 0*.*02*). Meanwhile, both infected, thermoregulating and uninfected locusts (CTR and CNTR) had significantly higher phagocytic activity on day 3 (*F =* 9.107, *P =* 0.006), day 4 (*F =* 9.225, *P =* 0.006), and day 5 (*F =* 6.858, *P =* 0.013). Thermoregulation did not alter the phagocytic activity within uninfected locusts but did so in the infected locusts. Indeed, thermoregulation enabled the infected locusts to engulf a significant number of beads even after fungal infection (Fig 9).

## Discussion

After pathogenic infection, many ectotherms such as reptiles and insects invoke a behavioral fever by seeking out higher temperatures, with expending additional efforts in thermoregulation compared to uninfected animals [53]. In our study, the *B*. *bassiana*-infected locusts elevated their body temperatures to as high as 30–32.6 °C, which was 1–3 °C higher than healthy conspecifics (Fig 1). The preferred thermal range is lower in this species than the fever temperature of 42–44 °C found in some acridid pests [33], suggesting that the locusts may not express a behavioral fever, as such, but are only thermoregulating during observation and experimental periods. This finding is similar to that reported in the acridid, *Chorthippus parallelus* (Zetterstedt), which regulates its body temperature up to 32–35 °C in response to *B*. *bassiana* infection [54]. These results suggest that the locusts may not be expressing a behavioral fever response to the fungal infection, but, instead, may be using the thermal behavior to extend their lives. The change in body temperature induced after fungal infection depends not only on the type of fungus and the insect species but also on environmental factors [21,26,33]. The capacity of insects to modulate the degree and duration of the fever response depends upon the nature of the pathogen, mainly its thermal sensitivity, and the severity of the pathogen challenge [33,55]. In one study, house flies infected with *B*. *bassiana* could tailor their patterns of fever by behavioral means, possibly limiting fever costs [55]. The increase in metabolic rate and other fitness costs related to fever temperatures can be substantial in species of Acrididae [28]. The lack of fever might be due to the physiological risks involved with raising temperatures above the normal range exceeding the advantages of the fever response. Even in the absence of fever, the degree of thermoregulation in *L*. *migratoria manilensis* was sufficient to influence and reduce growth in the pathogen because the optimum growth temperature of *B*. *bassiana* is 25°C [21,37].

Some studies have found that feeding in insects infected with *B*. *bassiana* or other fungi was affected, and that the reduction in food consumption may be attributed to effects the invading fungus has on the hormonal control of feeding [16,56]. Conversely, *L*. *migratoria migratorioides* infected by the entomopathogenic fungus, *M*. *anisopliae* var. *acridum*, showed a tendency to consume more than uninfected individuals [26]. Other studies, however, have found that feeding in infected insects decreased. *Lygus hesperus* Knight (Heteroptera: Miridae) infected with *B*. *bassiana* caused significantly more feeding damage that uninfected plant bugs [56]. In the present study, we found that infected locusts increased their time spent in Zone B, with shorter resting and feeding frequencies compared to healthy individuals (Figs 2 and 3). Differences in feeding pattern may also be dependent on host-pathogen interactions and the habitat conditions [57]. In addition, increased feeding by infected insects may be a response to nutrient deficiency, since entomopathogenic fungi remove nutrients from the host insect’s haemolymph [56].

We also observed that mortality decreased with increasing thermoregulation time (Fig 4), suggesting that behavioral thermoregulation in *L*. *migratoria manilensis* can reduce the efficacy of *B*. *bassiana* as a biopesticide. Insects exposed to 3 h/day of thermoregulation showed a great reduction in their mortality (43.34%) which was consistent with previous findings showing that access to a heat source for only 4 h/day was sufficient to greatly reduce mortality in infected *L*. *migratoria migratorioides* [26]. Similarly, mycosis by *B*. *bassiana* in grasshoppers was reduced by 46% reduction after only 1 h of basking, and as the basking time increased so did inhibition of fungal infection [58]. In addition, there is some evidence that the length of the interval between fungus inoculation and the onset of thermoregulation also plays an important role in increasing survival of infected hosts [59]. For instance, early access to thermoregulation was more effective in reducing locust mortality than when thermoregulation was delayed for 48 h [26]. We also noticed that when infected insects were allowed to thermoregulate 0 h and 24 h post-inoculation, mortality was reduced compared to locusts that were only allowed to thermoregulate 48 h post-inoculation (Fig 5). In the present study, thermoregulation helped all insects reduce their mortality rate by inhibiting pathogen growth. However, low mortality rates should not simply be interpreted as completely dependent on thermoregulation and behavioral fever extinguishing the potential of the entomopathogenic fungus, since locusts mortality still occurred under thermoregulation, although at a reduced rate, and continued until the end of the experiment. Furthermore, when thermoregulation was interrupted, the fungus continued to increase as did mortality in the insects (Fig 6). These results confirmed previous findings [26] showing that a thermoregulation regime helped infected locust delay mortality but did not eliminate infection. Mortality still occurred throughout the infected locusts’ lifetime after completion of the experiment.

Environmental temperature can significantly influence host-pathogen interactions by enhancing the host immune response [28,40]. Previous studies have shown an increase in phenoloxidase activity [49,39] associated with increases in temperature. Changes in haemocyte levels have also been observed in many insect-pathogen associations [38,60]. The present study determined the impact thermoregulation has on the host immune defense. A large decline in the haemocyte population was observed in infected, non-thermoregulating locusts on day 4 post-inoculation. Similar results were also observed in fifth instar silkworm (*Bombyx mori* L.) (Lepidoptera: Bombycidae) larvae with a reduction in total haemocyte count on day 4 following infection by *B*. *bassiana* [60]. We observed that, unlike *B*. *mori*, thermoregulation helped to increase concentration of haemocytes in infected locusts compared to healthy ones. Increases in haemopoiesis can occur in insects under immunological challenge [61]. This phenomenon may explain the rise in haemocyte number in infected locusts and the even higher numbers in those held under elevated thermal conditioning. In addition, high temperatures can also affect the growth of *B*. *bassiana* [37], allowing thermoregulating locusts to maintain higher haemocyte numbers by reducing the number of cells needed for immune defence activities such as nodulation and encapsulation.

We also assessed the influence of thermoregulation on the kinetics of *B*. *bassiana* blastospores in host haemolymph. Blastospores spread rapidly in the haemolymph of non-thermoregulating host and were numerous on day 4 in our study, which corresponded to the observed reduction in the host haemocyte population, findings that were in agreement with several previous studies [60]. No circulating blastospores were found in the haemolymph of thermoregulating insects after day 3 post-injection and few colony forming units were found when haemolymph of test insects was placed on selective media to detect non-visualized fungal units. No circulating blastospores were observed, as well, in locusts infected with *M*. *anisopliae var*. *acridum* that were exposed to thermoregulation conditions [38]. The absence of blastospores in the haemolymph of thermoregulating locusts may be the result of thermal inhibition of fungal growth, since the optimal temperature for *B*. *bassiana* growth is 25 to 30 °C [37].

In addition, the proportion of engulfed silica beads decreased in infected non-thermoregulating locusts as the infection progressed; whereas, infected, thermoregulating locusts maintained the same phagocytic activity level as uninfected locusts. A similar pattern had been previously reported in infected *L*. *migratoria migratorioides* [38]. While this study clarified how thermoregulation negatively affects the spread of fungal pathogens, such as *B*. *bassiana*, in insects, the mechanisms causing fungal suppression require further research.

In recent years, entomopathogenic fungi such as *B*. *bassiana* have been increasingly used as biopesticides for insect control, this is especially true in locust/grasshopper control [6]. However, several factors exist that interfere with the widespread acceptance of mycoinsecticides in pest management programs. These include their relatively slow mode of action in achieving lethality, a misconception of their perceived disruption of environmental conditions, and their susceptibility to host thermoregulatory behaviour. Previous studies have demonstrated that insects have evolved a variety of defense strategies such as behavioural fever against entomopathogenic fungi [24,25,28]. The behavioral changes resulting from fungal infection can have important implications in pest management strategies under field conditions. Thermoregulation and, to a larger extent, behavioural fever often cause insect body temperatures to increase to as high as 44 °C, which is substantially above the optimum temperature (20 to 30 °C) for germination and growth of most entomopathogenic fungi, including *B*. *bassiana* and *M*. *anisopliae* var. *acridum* [37,58]. We determined that *B*. *bassiana*-infected locusts not only elevated their body temperature and changed their basking and feeding behavior, but also increased their haemocyte count and maintained a high level of phagocytic activity, all of which adversely affect *B*. *bassiana*. These behavioural changes may occur in the field when *B*. *bassiana* is applied for locust control. Obviously, host thermoregulatory behaviour may play a major role in determining the control potential of entomopathogenic fungi, and the availability of days that allow behavioural fever may set temporal limits on the use of these fungi against locusts [26]. Therefore, to enhance the ability of *B*. *bassiana* to control locusts, appropriate strategies, in particular, inhibition of host behavioural thermoregulation, should be incorporated into the overall scheme for controlling the locusts. A better understanding of areas and times, including where and when locusts are most vulnerable to fungal infection, will help to improve the efficacy of mycoinsecticides [22,26]. Under field conditions, selecting favourable climatic condition to avoid high temperatures and targeting nymphal stages that have less effective thermoregulatory abilities, could help increase the efficacy of entomopathogenic fungi.

## Conclusion

Our study determined that thermoregulation can affect the virulence of *B*. *bassiana* used to control *L*. *migratoria manilensis*. Behavioural thermoregulation was found to increase the survival of infected locusts as long as they were allowed to bask, but did not completely relieve them of their infection. An increase in haemocyte number was also observed in infected thermoregulating locusts with a great level of phagocytic activity compared to infected non-thermoregulating locusts. These results demonstrate the role environmental conditions play in host-pathogen interactions and the need for selecting control strategies that can avoid host behavioral thermoregulation.
